# Influence of maternal overweight, obesity and gestational weight gain on the perinatal outcomes in women with gestational diabetes mellitus

**DOI:** 10.1038/s41598-017-00441-z

**Published:** 2017-03-22

**Authors:** Miao Miao, Mei Dai, Yue Zhang, Fang Sun, Xirong Guo, Guiju Sun

**Affiliations:** 1 Key Laboratory of Environmental Medicine and Engineering of Ministry of Education, and Department of Nutrition and Food Hygiene, School of Public Health, Southeast University, Nanjing, 210009 P.R. China; 2 State key Laboratory of Reproductive Medicine, Department of Nutrition, Nanjing Maternity and Child Health Care Hospital Affiliated to Nanjing Medical University, Nanjing, 210029 China; 3 Department of Epidemiology and Biostatistics, School of Public Health, Southeast University, Nanjing, 210009 P.R. China

## Abstract

To assess the associations between maternal body mass index (BMI) as well as gestational weight gain (GWG) and pregnancy outcomes in women with gestational diabetes mellitus (GDM). This is a retrospective analysis involving 832 nulliparous women complicated with GDM. Multivariate logistic and restricted cubic logistic regression were used to investigate the association of interest. Overall, 178 (21.4%) women were overweight or obese, and 298 (35.2%) exhibited excessive GWG. Compared with women of normal weight, high pre-pregnancy BMI resulted in a higher risk of cesarean section with an adjusted odds ratio of 1.95 (95% confidence interval being 1.29–2.96) for overweight group and 3.26 (1.57–6.76) for obese group. Similarly, the respective aORs were 4.10 (1.56–10.81) and 9.78 (2.91–32.85) for gestational hypertension, 2.02 (1.05–3.88) and 8.04 (3.46–18.66) for macrosomia, 2.14 (1.40–3.26) and 3.34 (1.69–6.60) for large for gestational age (LGA). Compared with adequate GWG, excessive GWG increased the incidence of cesarean section (1.60, 1.15–2.23) and macrosomia (1.94, 1.11–3.38), while inadequate GWG reduced the incidence of LGA (0.29, 0.17–0.51). High pre-pregnancy BMI and excessive GWG were associated with higher incidence of LGA, as well as other adverse outcomes in women with GDM. Narrower guidelines on GWG might offer extra safety benefit in gestational diabetic population.

## Introduction

Obesity has been designated as one of the most important global health threats worldwide, and its prevalence has been increasing among women of reproductive age^1^. Pregnant women constitute an important subpopulation with an elevated risk of obesity due to excessive weight gain. It has been shown that maternal obesity and excessive gestational weight gain (GWG) are associated with adverse obstetric and neonatal outcomes including spontaneous abortion, gestational diabetes mellitus (GDM), cesarean delivery, preeclampsia, neonatal macrosomia, and operative and anesthetic complications^2, 3^.

To support optimal pregnancy outcomes, the World Health Organization (WHO) recommended that the Institute of Medicine (IOM) develop guidelines for weight gain during pregnancy^4^. However, the IOM recommendations on gestational weight gain are based on pre-pregnancy BMI without taking into consideration different race/ethnicity, age, or existing pregnancy complications. Women with GDM are at increased risk of maternal and fetal complications including preeclampsia, preterm birth, caesarean section and delivery of large for gestational age (LGA) infants^5^. As obesity and GDM are frequently comorbid conditions, obesity and excessive gestational weight gain may compound these risks in GDM^6^. Because fat is an endocrine organ and interacts with diabetes, it is possible that the increased accumulation of fat has a differential effect on perinatal outcomes for women with GDM^7^.

Although several previous studies have examined the influence of high pre-pregnancy BMI and excessive GWG on perinatal outcomes such as cesarean delivery, preeclampsia and macrosomia, few were conducted in China, and mostly among non-GDM women^8, 9^. There is no direct evidence on the association between maternal pre-pregnancy BMI or GWG and the perinatal outcomes of GDM mothers, and remains unclear whether the latest 2009 IOM guidelines for pregnancy weight gain are applicable to GDM population, given the possible compound effect of obesity and gestational diabetes.

To address the abovementioned question, we designed a retrospective cohort study to associate maternal BMI and GWG with pregnancy outcomes in Chinese women with GDM and examine whether these narrower pregnancy weight gain recommendations are predictive of adverse perinatal outcomes in Chinese gestational diabetic population.

## Results

### Clinical characteristics of the study participants

The clinical characteristics of these 832 study participants are presented in Table 1. The median age of the 832 women at delivery was 31 years (range, 23 to 45 years), the median gestation week at delivery was 39.2 weeks, and the median GWG was 14.2 kg.

**Table 1:** Clinical characteristics of the study participants grouped by pre-pregnancy body mass index and gestational weight gain.

	Pre-pregnancy BMI (kg/m^2^)				GWG categories by IOM guidelines (kg)		
	Underweight	Normal weight	Overweight	Obese	Below	Within	Above
N (%)	96 (11.5)	558 (67.1)	134 (16.1)	44 (5.3)	187 (22.5)	352 (42.3)	293 (35.2)
Age, y							
<35	77 (11.1)	466 (67.2)	115 (16.6)	35 (5.1)	151 (21.8)	294 (42.4)	248 (35.8)
≥35	19 (13.7)	92 (66.2)	19 (13.7)	9 (6.5)	36 (25.9)	58 (41.7)	45 (32.4)
Parity							
Multiparous	11 (11.3)	64 (66.0)	16 (16.5)	6 (6.2)	27 (27.8)	42 (43.3)	28 (28.9)
Nulliparous	85 (11.6)	494 (67.2)	118 (16.1)	38 (5.2)	160 (21.8)	310 (42.2)	265 (36.1)
Height, cm	161.5 ± 4.5	161.4 ± 7.7	161.6 ± 4.4	161.2 ± 5.7	160.7 ± 4.4	161.4 ± 4.6	161.9 ± 9.7
Glycated hemoglobin, %	4.96 ± 0.28^*†^	5.04 ± 0.31^*†^	5.21 ± 0.31^‡§†^	5.43 ± 0.40^‡§*^	5.04 ± 0.34^||^	5.08 ± 0.33	5.11 ± 0.33^¶^
Gestational weeks#, weeks	39.3 ± 1.1	39.2 ± 1.4	39.2 ± 0.9	38.9 ± 2.1	39.0 ± 1.8	39.3 ± 1.3	39.3 ± 1.1
Birth weight#, g	3273 ± 341^§*†^	3291 ± 435^‡^	3485 ± 408^‡^	3470 ± 679^‡^	3206 ± 436^**||^	3413 ± 418^¶||^	3497 ± 433^¶**^
Pre-pregnancy BMI#††, kg/m^2^					21.60 ± 3.0^||^	21.65 ± 3.3^||^	22.43 ± 3.4^¶**^

Data presented as n (%) or mean ± standard deviation. BMI, body mass index; GWG, gestational weight gain; IOM, Institute of Medicine. ^*^P < 0.05 vs. overweight group; ^†^P < 0.05 vs. obese group; ^‡^P < 0.05 vs. underweight group; ^§^P < 0.05vs. normal weight group; ^||^P < 0.05 vs. excessive (‘above’) GWG group; ^¶^P < 0.05 vs. inadequate (‘below’) GWG group; # variance is non-homogeneous; ^**^P < adequate (‘within’) GWG group; ^††^The Correlation coefficient between Pre-pregnancy BMI and GWG is −0.283, P < 0.05.

According to the pre-pregnancy BMI, 96 women (11.5%) were underweight, 558 (67.1%) were of normal weight, 134 (16.1%) were overweight and 44 (5.3%) were obese (Table 1). The level of glycated hemoglobin was significantly higher in the overweight and obese groups than in normal weight and underweight groups (*P* < 0.05). In addition, birth weight was significantly higher in overweight or obese women than in underweight women (*P* < 0.05). There were no significant differences between the four pre-pregnancy BMI categories in maternal age, parity, height and gestational weeks (Table 1). Based on the IOM recommendations for GWG, 187 women (22.5%) exhibited inadequate GWG, 352 (42.3%) exhibited GWG within recommended levels, and 293 (35.2%) exhibited excessive GWG (Table 1). Pre-pregnancy BMI was significantly higher in women with excessive GWG (22.43 ± 3.4 kg/m^2^) than in women with adequate (21.60 ± 3.0 kg/m^2^) or inadequate (21.65 ± 3.3 kg/m^2^) GWG (*P* < 0.05). The level of glycated hemoglobin was significantly higher in women with excessive GWG than in women with inadequate GWG (*P* < 0.05). Birth weight was significantly higher in women with excessive GWG than in women with adequate or inadequate GWG (*P* < 0.05) (Table 1). The Correlation coefficient between Pre-pregnancy BMI and GWG is −0.283 (*P* < 0.01). There were no significant differences between the three GWG groups in maternal age, parity, height and gestational weeks.

Table 2 presents subgroup analysis of data for the various GWG categories according to pre-pregnancy BMI. Within underweight group, birth weight in the adequate GWG group was significantly higher than that in the inadequate GWG group and significantly lower than that in the excessive GWG group. Birth weight for normal women was significantly higher in adequate and excessive GWG than that in the inadequate GWG group. There was no significant difference between the three GWG groups in the birth weight of overweight and obese women. As the pre-pregnancy BMI increased, the total GWG and mean weight gain per week decreased (*P* < 0.05). In comparison to normal weight women, overweight women had a lower GWG and were more likely to gain weight above the IOM recommendations (OR, 2.48; 95% CI, 1.69–3.64). There were interaction effects of BMI * GWG on total gestational weight gain and mean weight gain per week (*P* < 0.05).

**Table 2:** Variation of birth weight, total GWG and mean weight gain per week with GWG category in each of the four pre-pregnancy BMI groups.

BMI	GWG category according to IOM	N (%)	Birth weight (g)	Total gestational weight gain (kg)	Mean weight gain per week (kg)
Underweight	Inadequate	25 (26.0%)	3076 ± 272^*^	10.49 ± 1.49^*^	0.27 ± 0.04^*^
	Adequate	48 (50.0%)	3278 ± 312^*^	15.10 ± 1.58^*^	0.38 ± 0.04^*^
	Excessive	23 (24.0%)	3478 ± 353^*^	24.43 ± 7.96^*^	0.62 ± 0.22^*^
Normal weight	Inadequate	136 (24.4%)	3184 ± 464^*^	8.95 ± 2.04^*^	0.23 ± 0.05^*^
	Adequate	244 (43.7%)	3441 ± 416^†^	13.86 ± 1.39^*^	0.35 ± 0.04^*^
	Excessive	178 (31.9%)	3479 ± 387^†^	20.18 ± 3.76^*^	0.51 ± 0.10^*^
Overweight	Inadequate	18 (13.4%)	3391 ± 203	5.26 ± 1.18^*^	0.13 ± 0.03^*^
	Adequate	44 (32.8%)	3418 ± 343	9.25 ± 1.42^*^	0.24 ± 0.04^*^
	Excessive	72 (53.7%)	3549 ± 471	16.47 ± 3.65^*^	0.42 ± 0.09^*^
obese	Inadequate	7 (15.9%)	3593 ± 514	2.97 ± 1.59^*^	0.08 ± 0.04^*^
	Adequate	17 (38.6%)	3382 ± 729	7.22 ± 1.55^*^	0.19 ± 0.04^*^
	Excessive	20 (45.5%)	3503 ± 707	13.13 ± 3.98^*^	0.34 ± 0.10^*^
Interaction effects^‡^			1.73	﻿3.23^§^﻿	﻿2.85^||^﻿

Data presented as n (%) or mean ± standard deviation. BMI, body mass index; GWG, gestational weight gain; IOM, Institute of Medicine. *P < 0.05 vs. both the other GWG groups; ^†^P < 0.05 vs^.^ inadequate GWG group; ^‡^the F-value for the interaction effects; ^§^interaction effect of BMI*GWG on total gestational weight gain, P < 0.05; ^||^interaction effect of BMI*GWG on mean weight gain per week, P < 0.05.

Tables 3 and 4 show the effects of pre-pregnancy BMI and GWG on pregnancy outcomes, expressed as the odds of each outcome occurring relative to that in women of normal weight or adequate GWG, respectively. In comparison to women of normal weight, overweight and obese women had a higher incidence of cesarean section, GHT, macrosomia and LGA (Table 3), with the association particularly strong for GHT (aOR, 9.78; 95% CI, 2.91.40–32.85; *P* < 0.01) in obese women group. Being underweight was associated with a lower incidence of both macrosomia (aOR, 0.20; 95% CI, 0.05–0.87; *P* = 0.03) and LGA (aOR, 0.31; 95% CI, 0.15–0.63; *P* < 0.01). Compared with GWG within the IOM recommendations, excessive GWG came with an increased incidence of cesarean section and infant macrosomia (Table 4), whereas inadequate GWG occurred with a lower incidence of LGA (aOR, 0.29; 95% CI, 0.17–0.51; *P* < 0.01). The association between pre-pregnancy BMI or GWG and LGA may be enhanced by the known cause that LGA is related to the gestational weeks. Therefore, the *P* value of LGA was lower than macrosomia’s.

**Table 3:** Effects of pre-pregnancy body mass index on pregnancy outcomes.

	Underweight (N = 96)			Normal weight (N = 558)	Over weight (N = 134)			obese (N = 44)		
	N (%)	AOR (95% CI)	P	N (%)	N (%)	AOR (95% CI)	P	N (%)	AOR (95% CI)	P
Cesarean section^a^	41 (42.7)	0.72 (0.45–1.15)	0.165	282 (50.5)	86 (64.2)	1.95 (1.29–2.96)	0.002	32 (72.7)	3.26 (1.57–6.76)	0.002
PPH^a^	12 (12.5)	0.79 (0.40–1.56)	0.501	88 (15.8)	31 (23.1)	1.60 (0.99–2.59)	0.057	6 (13.6)	0.81 (0.31–2.12)	0.671
Preterm delivery^b^	3 (3.1)	0.95 (0.27–3.30)	0.937	19 (3.4)	2 (1.5)	0.39 (0.09–1.70)	0.208	2 (4.5)	1.06 (0.22–5.08)	0.940
PPROM^b^	21 (21.9)	1.48 (0.86–2.54)	0.153	89 (15.9)	23 (17.2)	1.05 (0.63–1.75)	0.855	12 (27.3)	1.89 (0.90–3.95)	0.092
GHT^c^	1 (1.0)	0.48 (0.06–4.02)	0.499	10 (1.8)	8 (6.0)	4.10 (1.56–10.81)	0.004	5 (11.4)	9.78 (2.91–32.85)	0.000
Macrosomia^c^	2 (2.1)	0.20 (0.05–0.87)	0.031	41 (7.3)	15 (11.2)	2.02 (1.05–3.88)	0.034	12 (27.3)	8.04 (3.46–18.66)	0.000
SGA^b^	3 (3.1)	1.03 (0.29–3.62)	0.967	17 (3.0)	3 (2.2)	0.59 (0.17–2.13)	0.423	1 (2.3)	0.51 (0.06–4.22)	0.531
LGA^b^	10 (10.4)	0.31 (0.15–0.63)	0.001	132 (23.7)	47 (35.1)	2.14 (1.40–3.26)	0.000	17 (38.6)	3.34 (1.69–6.60)	0.001

AOR, adjusted odds ratio; CI, confidence interval; PPH, postpartum hemorrhage; PPROM, preterm premature rupture of membranes; GHT, gestational hypertension; SGA, small for gestational age; LGA, large for gestational age. AORs are presented relative to the normal weight group.

^a^Adjusted for maternal age, gestational weeks, gestational weight gain and birth weight; ^b^adjusted for maternal age and gestational weight gain; ^c^adjusted for maternal age, gestational weeks and gestational weight gain.

**Table 4:** Effects of gestational weight gain on pregnancy outcomes.

	Inadequate GWG (N = 187)			Adequate GWG (N = 352)	Excessive GWG (N = 293)		
	N (%)	AOR (95% CI)	P	N (%)	N (%)	AOR (95% CI)	P
Cesarean section^a^	94 (50.3)	1.09 (0.75–1.59)	0.646	170 (48.3)	177 (60.4)	1.60 (1.15–2.23)	0.005
PPH^a^	26 (13.9)	1.07 (0.63–1.81)	0.800	51 (14.5)	60 (20.5)	1.44 (0.94–2.19)	0.094
Preterm delivery^b^	9 (4.8)	1.60 (0.65–3.93)	0.310	11 (3.1)	6 (2.0)	0.63 (0.23–1.73)	0.369
PPROM^b^	36 (19.3)	1.26 (0.79–2.01)	0.326	58 (16.5)	51 (17.4)	1.01 (0.66–1.54)	0.965
GHT^c^	3 (1.6)	0.55 (0.15–2.06)	0.374	10 (2.8)	11 (3.8)	1.23 (0.50–2.98)	0.655
Macrosomia^c^	7 (3.7)	0.55 (0.23–1.35)	0.194	24 (6.8)	39 (13.3)	1.94 (1.11–3.38)	0.020
SGA^b^	8 (4.3)	1.59 (0.64–4.50)	0.339	9 (2.6)	7 (2.4)	0.78 (0.29–2.08)	0.615
LGA^b^	17 (9.1)	0.29 (0.17–0.51)	0.000	92 (26.2)	97 (33.1)	1.31 (0.92–1.85)	0.133

AOR, adjusted odds ratio; BMI, body mass index; CI, confidence interval; GWG, gestational weight gain; PPH, postpartum hemorrhage; PPROM, preterm premature rupture of membranes; GHT, gestational hypertension; SGA, small for gestational age; LGA, large for gestational age. AORs are presented relative to the adequate GWG group.

^a^Adjusted for maternal age, gestational weeks, pre-pregnancy body mass index and birth weight; ^b^adjusted for maternal age and pre-pregnancy body mass index; ^c^adjusted for maternal age, gestational weeks and pre-pregnancy body mass index.

**Figure 1:**
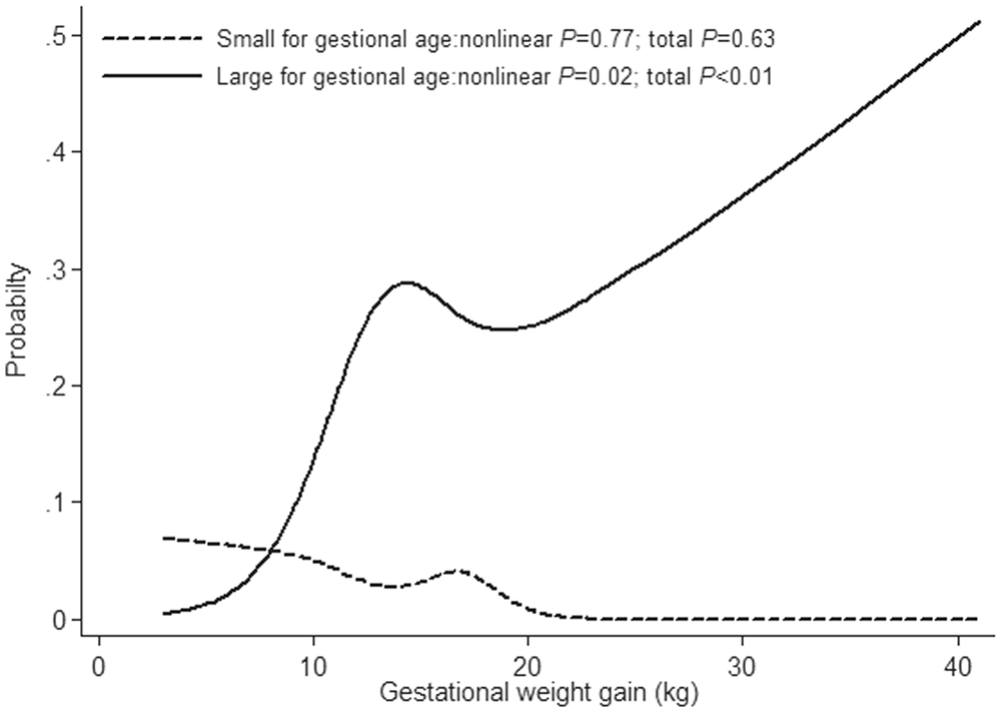
Probabilities* of delivering an infant too small or too large for gestational age by gestational weight gain in women with a normal† body mass index (BMI), Nanjing, China. *Logistic regression analyses with cubic splines adjusted by maternal age, parity and gestational weeks. ^†^BMI ≥ 18.5 but <23.9 kg/m^2^.

For each maternal pre-pregnancy BMI category we examined the association between gestational weight gain and the probability of delivering an infant too small or too large for gestational age in women with GDM. The greater the gestational weight gain, the higher the risk of delivering an infant too large for gestational age (*P* < 0.01) (Fig. 1). For women with normal BMI, the risk of having an infant too small for gestational age exceeded the risk of having one too large for gestational age with pregnancy weight gains up to only 8.0 kg. We also observed similar association for other pre-pregnancy BMI groups, but due to diminished statistical power for the outcomes of interest, these results were not presented.

## Discussion

Maternal, perinatal and neonatal complications are strongly associated with GDM. The incidence of GDM in China has increased since 2000 and this has become an important public problem in China^10, 11^. A Chinese national survey had reported prevalence of the IADPSG criteria-defined GDM of 14.7% in 2004–2009^12^. This incidence of GDM is similar to other studies in Asian populations^13^, but higher than the incidence of GDM in the United Kingdom (3.5%)^14, 15^ and the United States (8.6%)^16^. Incidence of GDM seems to depend on factors such as ethnicity and geographical regions^15^. In 2007 through 2008, nearly 60% of reproductive-age American women were reported to be overweight or obese, with the prevalence of overweight or obesity reported at approximately 21.4% in our study. Although the incidence of obesity is lower in Chinese and Asian women compared with other ethnicities^17, 18^, previous studies have shown that Asians have a much higher risk of GDM, even at a very low BMI^19^.

Women with GDM are at risk of maternal and neonatal complications in pregnancy, and being overweight or obese with excessive gestational weight gain appears to compound this risk^7^. The main findings of the present study are that, compared to women of normal weight, overweight and obese women with GDM had a higher incidence of cesarean section, GHT, macrosomia and LGA, while underweight women had a lower incidence of both macrosomia and LGA. Furthermore, compared with GWG within the IOM recommendations, excessive GWG increased the incidence of cesarean section and infant macrosomia, while inadequate GWG decreased the incidence of LGA. Although most studies addressing the effects of maternal BMI on adverse outcomes include women with GDM^20–24^, a few have reported these associations in overweight or obese women with normal glucose tolerance^8, 9, 25^. Scant data exist that demonstrate the interaction between high maternal pre-pregnancy BMI, gestational weight gain and perinatal outcomes in women with GDM.

In our study, total GWG and mean weight gain per week decreased with increasing pre-pregnancy BMI. These data conformed with previous reports that women in the highest BMI category gained less weight than those in the lowest category among nondiabetic or mixed populations of pregnant women^26, 27^. Nonetheless, although total GWG was lower in overweight/obese women, the proportion of women with a GWG that exceeded the IOM recommendations was higher in overweight women than in women of normal or obese BMI. Though these results cannot be wholly explained by the current analysis, they may highlight a bias in the emphasis on management of diabetes during pregnancy among care providers, where by the message relating to weight management is reinforced more vigorously in obese rather than overweight women.

The odds of cesarean section were increased in both overweight (OR 1.95) and obese women (OR 3.26) as well as in women with excessive GWG (OR 1.60) in the present study. Researchers agree that there is a substantial relationship between BMI and cesarean delivery, but the relationship between maternal weight gain and cesarean delivery has recently become a controversial subject. For example, Ines Gante *et al*.^28^ found that excessive GWG is significantly associated with an additional risk of cesarean section (OR 1.3) even after adjusting for other factors including birthweight, while others have concluded that GWG does not have a significant influence on the occurrence of cesarean delivery^29^. Consistent with previous studies^28^, we found that women in the excessive GWG group had a higher likelihood of emergency cesarean delivery than those in the adequate GWG group.

Our study showed that the odds of GHT were much higher in women who were overweight or obese before pregnancy (ORs 4.10 and 9.78, respectively). The observations concur with data from several previous investigations on those pregnant women with or without GDM. Gaillard *et al*.^30^ determined the odds of GHT to be 3-fold higher in women with a high pre-pregnancy BMI. Tanaka T^31^ reported that GHT was associated with an increased pre-pregnancy BMI and high GWG. Although we initially noted a increased incidence of GHT in women with excessive GWG (3.8% vs 2.8%), these findings did not persist in the multivariate logistic regression analysis (OR, 1.23; 95% CI, 0.50–2.98).

After adjustments were made for possible confounding factors, the odds of LGA and macrosomia were calculated to be higher in women who were overweight or obese before pregnancy. In addition, the odds of LGA and macrosomia were reduced in underweight women (ORs 0.20 and 0.31, respectively). We also observed that excessive GWG increased the incidence of infant macrosomia, while inadequate GWG decreased the incidence of LGA. Consistent with these findings, Mary HB *et al*.^27^ recently reported higher adjusted odds of LGA for overweight and obese women and for women with excessive GWG among women with and without GDM. Our stratification of underweight, normal weight, overweight and obese groups of women also allowed us to detect decreased risk of LGA and macrosomia for underweight compared with normal-weight women. Futhermore, we did not find increased odds of SGA in underweight and inadequate GWG group. Based on these findings, we speculate that GDM women with lower pre-pregnancy BMI or lower GWG are somewhat protected against LGA. Perhaps a weight gain less than IOM recommended weight gain would be adequate for women with GDM.

In our study, GWG was inversely correlated with the risk of having an SGA baby and directly correlated with the risk of LGA. A weight gain of 8.0 kg in women with a normal BMI (18.5–23.9 kg/m^2^) was proved to be optimal. The IOM recommends a gain of 11.5–16.0 kg in this group of women. Based on these data, we would recommend that lower thresholds for weight gain may improve outcomes in women with GDM. Further research is required to determine what range of gestational weight gain minimized the risk of having infants too small or large for gestational age among gestational diabetic women with different pre-pregnancy BMI. Going forward, the specific actions recommended by the IOM in the new guidelines should also include recommendations for the populations of diabetic women.

There are several limitations to the current study. This was an observational, retrospective, single-center study, hence selection and information bias cannot be ruled out, and the enrolled cohort may not be representative of the general population in China or beyond. We only had access to mode of delivery, but had no access to the type of caesarean. Therefore the influence of maternal pre-pregnancy weight and gestational weight on different types of caesarean may be different. Some outcomes such as “Preterm delivery” and “GHT” are very rare. Thus, the results may not be too reliable. Additionally, diet and physical activity may have important influences on GWG, thus it was not possible to discriminate the associations between maternal weight/GWG and perinatal outcomes from any associations between diet/physical activity and these perinatal outcomes. Women with GDM received intervention during the third trimester (dietary control of energy intake plus insulin therapy if required), which may have influenced the associations between GWG and perinatal outcomes.

Despite these limitations, our study has several strengths. Acknowledging the limitations associated with observational study design and associated influence of measured and unmeasured covariates, we have used adjusted multivariate regression analysis to provide convincing and strong associations of pre-pregnancy BMI and GWG with perinatal outcomes in women with gestational diabetes mellitus. We have also used strong, validated classification systems for the definition of GWG and BMI. Moreover, cubic spline logistic regression analysis was performed to examine potential nonlinear associations between small or large size for gestational age and gestational weight gain in each maternal BMI category. Knowledge of this information may help us to better understand whether IOM recommendations are applicable to Chinese women with GDM.

In summary, our data suggest that high pre-pregnancy BMI and excessive GWG are associated with higher incidences of LGA, as well as other adverse outcomes in Chinese women with GDM. Narrower guidelines for GWG might be safer and beneficial in a gestational diabetic population. Further research should set out to determine the optimal range of GWG in order to minimize the risk of adverse perinatal outcomes. Based on this, helping overweight or obese women lose weight before pregnancy and/or controlling weight gain during pregnancy should be able to materialize the potential benefit in terms of maternofetal outcomes for these GDM women and their offspring, especially in Asian countries which have a much higher risk of GDM even at a very low BMI.

## Methods

### Ethics

This investigation conforms to the principles outlined in the Declaration of Helsinki. This study was approved by the Ethics Committee of The Hospital of Maternity and Child Health Care, Nanjing, China, No. 201372. All patients provided written informed consent prior to participation in the study protocol.

### Study participates

The present study is a retrospective analysis of data collected prospectively from women who delivered single live babies at the Nanjing Maternity and Child Health Care Hospital affiliated to Nanjing Medical University between December 2013 and December 2014. During the study period, the total number of live births was 15,498. Of these live births, 1516 (9.8%) mothers developed GDM. All women confirmed with GDM were invited to participate in the trial unless they had one or more of the following exclusion criteria: an incomplete dataset available; multiple pregnancy; a history of hypertension, diabetes, heart disease, hepatitis, chronic renal disease or other systemic disease. Finally, a total of 832 women were included in this study. All data on the pregnancies complicated by GDM were manually pulled from the Mother’s Medical Records which were routinely updated during pregnancy. Oral glucose tolerance tests (OGTT) were measured by a 1 step approach between 24th and 28th weeks of gestation. A 75 g glucose load was administrated after fasting glucose and plasma glucose levels were measured after 1 and 2 h. GDM was diagnosed if either of the glucose values fell at or above the specific glucose threshold (≥5.1 mmol/L for fasting, ≥10.0 mmol/L at 1 h, ≥8.5 mmol/L at 2 h).

The maternal age, glycated hemoglobin at diagnosis, gestational week at delivery, parity, maternal body mass index (BMI), maternal weight gain, birth weight and the glucose levels of GDM patients were recorded.

Body mass index (BMI) was calculated by dividing pre-pregnancy weight in kilograms by the square of height in meters. Pre-pregnancy BMI was categorized as underweight (BMI < 18.5 kg/m^2^), normal weight (18.5–23.9 kg/m^2^), overweight (24.0–27.9 kg/m^2^), or obese (≥28.0 kg/m^2^) using the standard defined by the Working Group on Obesity in China^32^. Weight gain of mothers during pregnancy was calculated as the difference between pre-pregnancy and delivery weight. Adequacy of GWG was defined according to the Chinese maternal pre-pregnancy BMI status and the 2009 IOM GWG recommendations: 12.5–18 kg (pre-pregnancy BMI < 18.5 kg/m^2^), 11.5–16 kg (BMI 18.5–23.9 kg/m^2^), 7–11.5 kg (BMI 24.0–27.9 kg/m^2^), and 5–9 kg (BMI ≥ 28 kg/m^2^)^4^. We used the translation of US IOM GWG recommendations because no official recommendation exists in China.

### Obstetrical and neonatal outcomes

We considered the risks of caesarean section, postpartum hemorrhage, preterm birth (preterm delivery), preterm premature rupture of membranes, pregnancy-induced hypertension, macrosomia, small for gestational age (SGA) infant, large for gestational age (LGA) infant as pregnancy complications and pregnancy outcomes. Postpartum hemorrhage (PPH) was defined as blood loss ≥500 mL within 24 hours of delivery, as recorded on the delivery summary by a nurse or doctor, regardless of the mode of delivery. Preterm delivery was defined as gestational weeks of delivery <37 weeks. Preterm premature rupture of membranes (PPROM) was defined as a spontaneous rupture of membranes before the onset of labor and before 37 weeks of gestation. Pregnancy-induced hypertension (PIH) was diagnosed by a systolic blood pressure ≥140 mmHg or diastolic blood pressure ≥90 mmHg in the 3rd trimester or using antihypertensive drugs. Macrosomia was defined as birth weight ≥4000 g. A SGA infant was defined as an infant having a standardized birth weight <10th percentile, whereas a LGA infant was defined as an infant having a standardized birth weight >90th percentile.

All women with GDM received a recommendation for their diet during pregnancy. For those women who had poor glycemic control despite dietary and lifestyle intervention, insulin therapy was given. The targets for insulin treatment are fasting glucose level within 3.3–5.6 mmol/L and two hour post-prandial ≤6.7 mmol/L.

### Statistical analyses

Statistical analyses were performed using the SPSS software package (version 18.0; SPSS Inc, Chicago, IL, USA). Data are expressed as mean ± standard deviation, median (range) or number (percentage). Statistical comparisons of categorical data were made using the chi-square (χ^2^) test. All continuous data with homogeneity of variance were compared by one way ANOVA with LSD, or by nonparametric K-W test followed by pairwise comparisons. The P value was adjusted by Bonferroni correction to counter the multiple comparisons between different groups. Adjusted *P* value < 0.05 was considered statistically significant.

Multivariate logistic regression analysis was performed to identify associations between pre-pregnancy BMI or GWG and various pregnancy outcomes (cesarean section, PPH, preterm birth, PPROM, PIH, SGA and LGA), after adjustment for potential confounding variables including age, gestational weeks, birthweight and pre-pregnancy BMI/GWG. Adjusted odds ratios (aORs) and 95% confidence intervals (CIs) were calculated for each pregnancy outcome, in order to determine the odds of high or low pre-pregnancy BMI relative to normal pre-pregnancy BMI, and of inadequate or excessive GWG relative to adequate GWG. All ORs were adjusted for maternal age, maternal height and pre-pregnancy BMI or GWG (as appropriate). Additional adjustments were made, as follows: gestational weeks and birth weight for cesarean section and PPH; and gestational weeks for GHT, SGA and LGA.

Restricted cubic spline logistic regression analysis^33, 34^ was performed to fit nonlinear curves smoothly using Stata Version 12 (Stata Corp LP, College Station, TX, USA). This was done to examine potential nonlinear associations between small or large size for gestational age and gestational weight gain in each maternal BMI category. Maternal age, parity and gestational weeks were adjusted in the multivariate model. Optimal weight gains were determined from the intersection on the regression graph of maternal weight gain and the probability of delivering an infant too small or too large for gestational age.
